# Efficacy and clinical outcomes of proximal splenic artery embolization in patients with chemotherapy induced thrombocytopenia

**DOI:** 10.1007/s00423-026-04144-w

**Published:** 2026-08-26

**Authors:** Daniel Körfer, Madelon Dijkstra, Brian Schiro, Constantino Pena, Gina Landinez, Govindarajan Narayanan, Alex Powell, Ripal Gandhi

**Affiliations:** 1 Department of Interventional Radiology, Miami Cardiac and Vascular Institute, Baptist Health South Florida, Miami, FL USA; 2 Department of Vascular and Endovascular Surgery, Heidelberg University Hospital, Im Neuenheimer Feld 420, Heidelberg, 69120 Germany; 3 Department of Interventional Oncology, Miami Cancer Institute, Baptist Health South Florida, Miami, FL USA; 4 Department of Radiology and Nuclear Medicine, Cancer Center Amsterdam, Amsterdam UMC, Location VUmc, Amsterdam, The Netherlands; 5 Department of Interventional Radiology, Herbert Wertheim College of Medicine, Florida International University, Miami, FL USA

**Keywords:** Splenic Artery, Therapeutic Embolization, Chemotherapy, Thrombocytopenia

## Abstract

**Purpose:**

This retrospective study aims to investigate efficacy and clinical outcomes of proximal splenic artery embolization (PSAE) in patients with chemotherapy induced thrombocytopenia (CIT).

**Methods:**

PSAE was performed in thirteen patients with CIT (6:7, M:F; median age: 55y; range: 39-76y) with underlying malignancy (colorectal carcinoma: *n* = 6; pancreatic carcinoma: *n* = 3; other: *n* = 4). Median platelet counts were calculated prior to chemotherapy, at nadir resulting in chemotherapy discontinuation, prior to SAE, immediately and at the maximum following SAE. Additionally, the time until re-initiation of chemotherapy was calculated.

**Results:**

Technical success was 100%, with no adverse events or 30-day mortality. Vascular plugs (*n* = 9) and coils (*n* = 11) were the most frequently used devices. Median platelet count pre-chemotherapy was 201 × 10(9)/L (range: 100–423 × 10(9)/L), platelet count nadir was 45 × 10(9)/L (range: 21–69 × 10(9)/L), and pre-SAE platelet count was 82 × 10(9)/L (range: 45–171 × 10(9)/L). After proximal SAE, median platelet count increased significantly (110 × 10(9)/L; range: 68–169 × 10(9)/L) compared to pre-procedure platelet count (*p* = 0.027). Median platelet count at the maximum at follow-up was 191 × 10(9)/L (range: 31–518 × 10(9)/L). Chemotherapy was re-initiated after a median of 15 days (range: 7-50d).

**Conclusion:**

Proximal SAE is a safe and effective treatment option in patients with CIT, with potential benefits in early re-initiation of chemotherapy. Further studies should be conducted to evaluate the longer-term effects of the procedure, further identify eligible patients, and compare to distal SAE.

## Introduction

Chemotherapy induced thrombocytopenia (CIT) is a common adverse event in cancer patients undergoing cytotoxic therapy. The condition is generally defined by a platelet count below 100 × 10(9)/L and results in reduced chemotherapy dosages, bleeding complications and unfavorable oncological outcomes [1].

Thrombocytopenia in patients with cancer can be caused by the primary disease, however, chemotherapy causing myelotoxicity is the most common reason. The incidence of CIT varies greatly depending on type of cancer, treatment, age, and comorbidities. In a recent retrospective analysis, the estimated incidence of thrombocytopenia was 28% for hematological cancers and 13% for solid tumors within three months after chemotherapy initiation [2]. Hematological cancers seem more prone to CIT, although such patients usually have lower platelet counts at the beginning of therapy. In a large US database analysis comparing the thrombocytopenic effect of different therapeutic agents, CIT was most prevalent in gemcitabine-based (23.2%), followed by platinum-based (19.9%), anthracycline-based (10.1%) and taxane-based (4.3%) agents [3].

CIT presents an increased risk of bleeding, often resulting in the reduction or discontinuation of chemotherapy. A high level of attention and monitoring is required, given the fact that spontaneous hemorrhage is the leading cause of death for a platelet count < 5 × 10(9)/L [4]. CIT has a negative impact on treatment outcome in affected patients by increasing the relative dose intensity and the therapeutic effect [5, 6].

Management of CIT focuses on prevention and control of major bleedings. There are several drug therapy approaches to increase the platelet count. Oprelvekin (IL-11) has shown to increase platelets in a series of clinical trials, although described side effects (including atrial arrhythmias) limited clinical efficacy [7, 8]. Thrombopoietin (TPO) replacement therapy with agents such as Romiplostim, Eltrombopag and Avatrombopag aim to promote the production of megakaryocytes and increase platelet formation with encouraging early results in clinical trials, but was limited due to cross-reactivity of anti-TPO autoantibodies with endogenous TPO causing drug-induced thrombocytopenia [9–11]. Hence, platelet transfusion appears to be the most effective medical therapy for severe thrombocytopenia [12].

Aiming to therapeutically reduce splenic blood flow, splenic artery embolization (SAE) is an additional treatment option for patients with CIT. The interventional procedure is a safe alternative to splenectomy and is an established technique in splenic trauma management and for treatment of thrombocytopenia and portal hypertension [13–15]. Regardless of the indication, the splenic artery can be embolized proximally, distally or in combination. With the idea of decreasing perfusion pressure to achieve hemostasis, proximal splenic artery embolization (PSAE) is generally used for trauma control by simultaneously decreasing the risk of infarction and abscess formation by preserving the collateral network [16, 17]. The goal of distal splenic embolization (DSE), also described as partial SAE, is to eliminate blood supply of 30–70% of the splenic parenchyma for improvement of hematologic value [18]. DSE (or partial SAE) is considered a safe intervention able to increase platelet counts across a range of malignancies and result in the re-initiation of chemotherapy [19, 20]. Studies regarding PSAE are limited in number of treated patients, but they show similar results in terms of efficacy [15, 21]. In addition, PSAE showed advantages in terms of safety profile and length of stay compared to DSE in the management of hypersplenism [22, 23]. Particularly noteworthy in this context is post-embolization syndrome, which can occur following SAE. Currently, post-embolization syndrome has no distinct definition. It comprises symptoms such as fever, nausea, vomiting, left upper quadrant pain and pleural effusion [14, 24]. These symptoms usually occur within the first 72 h after solid organ embolization, mainly driven by tissue infarction and necrosis, which leads to release of inflammatory mediators and vasoactive substances [25]. Usually, symptoms are self-limiting, and prolonged presence may indicate the manifestation of a systemic infection, requiring immediate alternate management beyond symptomatic therapy. In the few studies describing proximal and distal SAE, proximal embolization was found to be better tolerated in terms of the mentioned symptoms and other complications, such as splenic abscess [22, 23, 26, 27]. For this reason, the PSAE approach was preferred in clinical practice and subsequently in this study, raising the question of whether proximal SAE is superior to the distal SAE in the overall outcome.

The purpose of this study is to analyze technical safety and clinical outcome of proximal splenic artery embolization to alleviate CIT, allowing patients with cancer to resume chemotherapy.

## Materials and methods

This single-center, IRB-approved study retrospectively analyzed patients with CIT undergoing PSAE and adhered to the ‘Strengthening the Reporting of Observational studies in Epidemiology’ (STROBE) guidelines [28].

### Patients selection and data collection

A retrospective analysis of patients with CIT who were consented for PSAE was performed. Baseline patients’ characteristics, including sex, age, cancer type, chemotherapeutic agent and number of cycles were reviewed and obtained from electronic patient files. All patients were discussed in a multidisciplinary setting prior to treatment and patients were referred to the department of Interventional Radiology by their medical oncologists. Thrombocytopenia was defined as a platelet count below 100 × 10(9)/L.

### Patient characteristics

Between October 2016 and October 2023, PSAE was performed in thirteen patients with CIT (6 men, 7 women). The median age was 55 years (range 39–76 years). The underlying malignancies were colorectal carcinoma (*n* = 6), pancreatic carcinoma (*n* = 3), esophageal carcinoma (*n* = 2), gastric carcinoma (*n* = 1) and endometrial carcinoma (*n* = 1). A combination of folinic acid, fluorouracil, oxaliplatin and/or irinotecan were the most frequently used chemotherapeutic agents. Individual chemotherapy regimen are displayed in Table 1. Median cycles of chemotherapy was eight (range 2–14 cycles) before PSAE.

**Table 1:** Patient characteristics and chemotherapy regimen

Patient	Sex	Age (years)	Primary cancer type	Chemotherapeutic agent	Number of chemotherapeutic cycles
#1	Female	47	Colorectal	FOLFOX + Bevacizumab	14
#2	Female	55	Endometrial	DOXIL + Bevacizumab	2
#3	Female	72	Gastric	FOLFOX + Trastuzumab	12
#4	Male	57	Esophageal	FOLFOX	8
#5	Male	65	Pancreatic	Gemcitabine + Paclitaxel	8
#6	Female	39	Colorectal	XELODA + Bevacizumab	8
#7	Female	40	Colorectal	FOLFOX + Bevacizumab	13
#8	Male	74	Pancreatic	FOLFIRINOX	6
#9	Male	67	Pancreatic	FOLFIRINOX	6
#10	Female	49	Colorectal	FOLFOX + Bevacizumab	6
#11	Male	53	Esophageal	FOLFOX + Nivolumab	14
#12	Male	76	Colorectal	FOLFOX + Bevacizumab	2
#13	Female	50	Colorectal	FOLFOX + Bevacizumab	10

### Proximal splenic artery embolization procedure

PSAE has become the standard practice for CIT in the authors’ institution. Reasons for abandoning splenectomy include the minimally invasive approach of embolization, faster recovery, and ability to continue or re-initiate systemic therapy faster. Choice of proximal SAE was based on local expertise and at the discretion of the interventional radiologist. Intravenous antibiotics were administered to all patients before and after the PSAE. All patients received pneumococcal vaccine prior to the procedure in accordance with institutional standard procedures. None of the patients necessitated platelet transfusion prior to the PSAE procedure. Conscious sedation was provided by the physician(s) performing the procedure. Interventional goal during PSAE was to embolize ⅔ to ¾ of the splenic parenchyma. Celiac and splenic artery angiogram was performed to assess the proximal splenic artery. Embolization of the splenic artery was performed distal to the origin of the dorsal pancreatic and pancreatica magna arteries using coils and vascular plugs. Information regarding procedural characteristics, including choice of embolization agent, was collected from the procedural notes in the electronic patient files. Post-embolization angiogram was conducted to confirm the comprehensive embolization, assess blood vessels, and examine for any immediate post-procedural adverse events. Patients were moved to the recovery area for a minimum of 4 h, and if deemed necessary, admitted for overnight observation to ensure there is no bleeding from the arterial access site (increased risk given thrombocytopenia) as well as to monitor pain and other adverse events following the procedure. Follow-up was conducted by the medical oncologists, with routine laboratory tests, including a complete blood count, every 1–3 weeks. The platelet level to reinitiate chemotherapy dependent on the judgment of the individual medical oncologist.

### Objectives and statistical analysis

The primary outcome measures of the study were efficacy using short- and long-term platelet count and safety, including documented adverse event and 30-day mortality. Platelet count was described using median and range (minimum/maximum). Median platelet counts were calculated prior to chemotherapy, at nadir resulting in chemotherapy discontinuation, prior to SAE, immediately and at the maximum following SAE. The platelet count at distinct time intervals is displayed in a linear graph. Comparison of median platelet values was performed with two-sided Wilcoxon signed-rank test analysis. Adverse events were assessed using Common Terminology Criteria for Adverse Events (CTCAE) 5.0 [29]. Typical mild post-embolization symptoms (such as nausea and mild abdominal pain) were not specifically evaluated. Secondary outcome measures were technical success, pre- and post-procedural splenic volume, length of hospital stay and time to resume chemotherapy. Technical success was defined as successful embolization of the proximal splenic artery. The splenic (enhanced) volumes were calculated by using volume-rendering software (MIM v7.1.6, MIM Software Inc., Cleveland, Ohio, USA) for computed tomography (CT) or magnetic resonance imaging (MRI) at baseline before the procedure and based on the first available appropriate imaging after PSAE. Splenic comparison of the mean volumes was performed with paired t-test analysis. The splenic infarction rate was calculated by using the following formula: (pre-PSAE enhanced volume – post-PSAE enhanced volume)/pre-PSAE enhanced volume. A significance level of less than 0.05 was considered to represent statistical significance. Data analysis was performed using Excel (Microsoft), SPSS^®^ Version 28.0 (IBM^®^, Armonk, New York, USA) and R version 4.2.1. (R Foundation, Vienna, Austria), and results were tabulated and displayed in graphs.

## Results

### Adverse events

No adverse events CTCAE Grade 3 or higher occurred. The 30-day mortality rate was 0%. Depending on the scheduled time of the procedure, all patients stayed until the end of the day or overnight. All patients were referred back to their medical oncologist for further follow-up.

### Efficacy

**Fig. 1:**
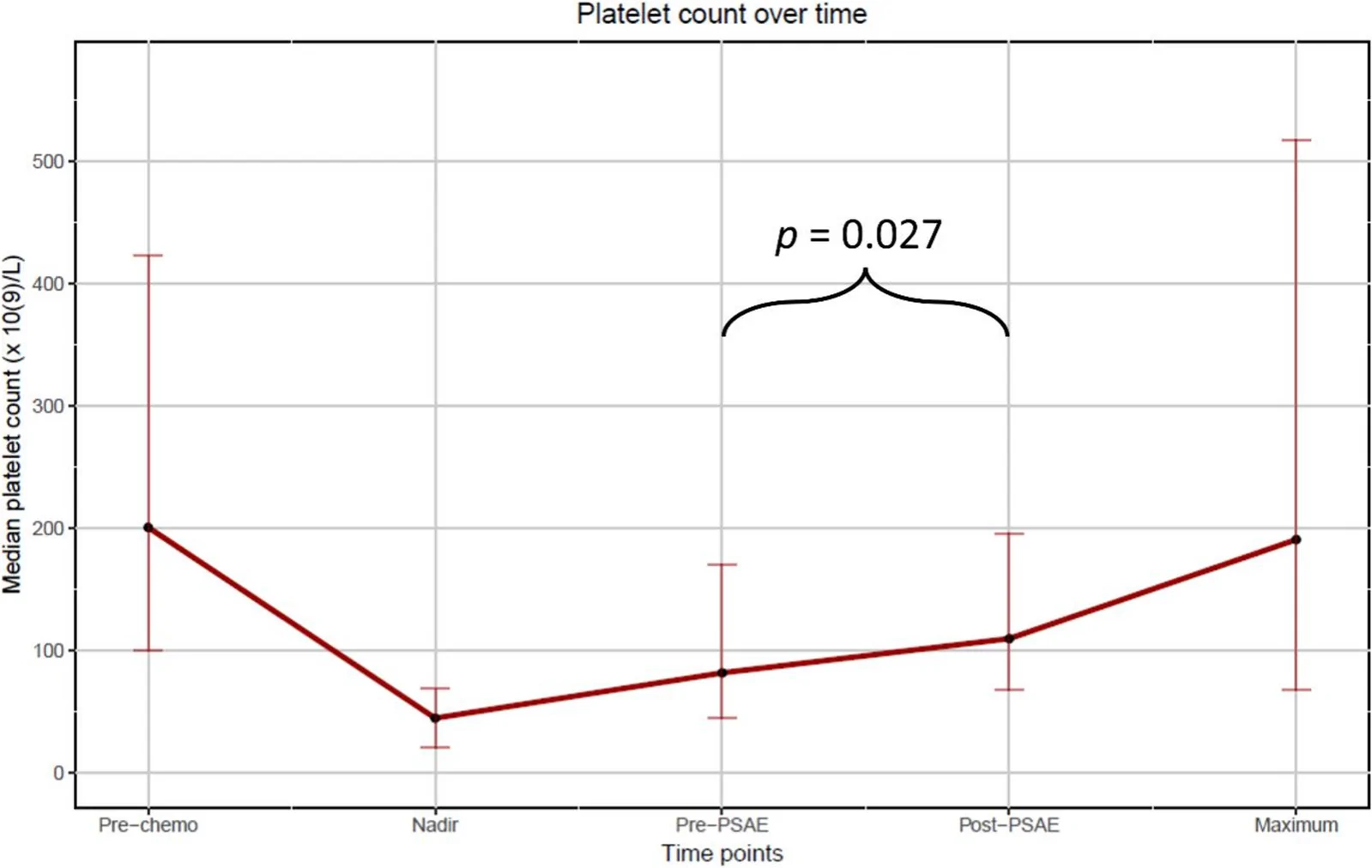
Platelet count pre-chemotherapy, nadir, before PSAE, after PSAE and at the maximum at follow-up (median and range [minimum/maximum])

**Fig. 2:**
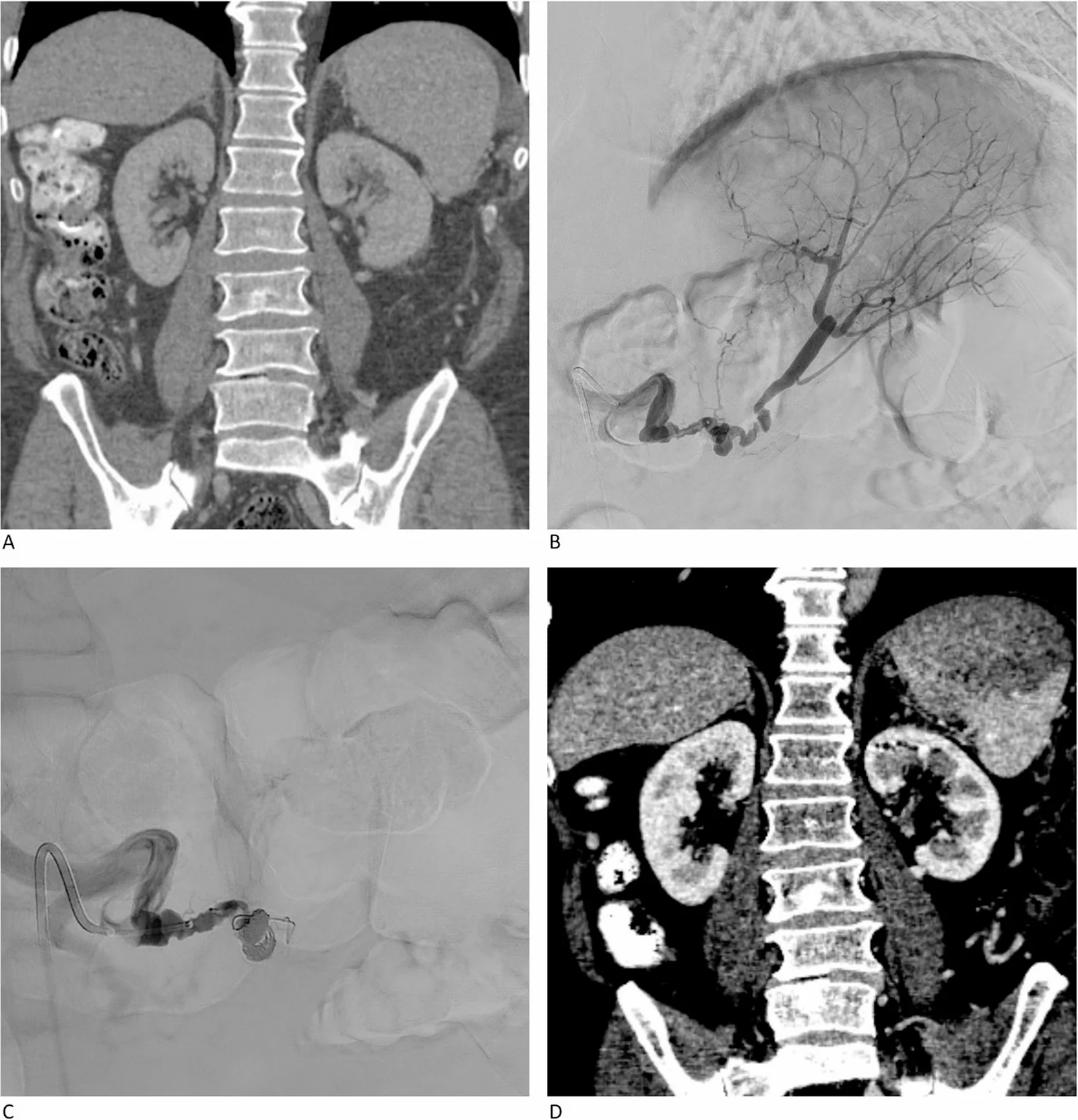
Example case of a 47-year-old man with pancreatic cancer undergoing PSAE. The patient developed CIT under chemotherapy with FOLFIRINOX, with a nadir platelet count of 67 × 10(9)/L. (**A**) Pre-procedural CT angiogram showing an enlarged spleen (pre-SAE volume 409.1 cm3). (**B**) Intra-procedural angiogram showing the splenic artery and the enlarged spleen. (**C**) Intra-procedural angiogram after placement of coils in the proximal splenic artery. (**D**) Post-procedural CT angiogram at 2-month follow-up showing splenic infarction (post-SAE enhanced volume 126.7 cm3, splenic infarction rate 69.0%). Post-SAE platelet count increased to 190 × 10(9)/L 55 days after the procedure

Technical success was 100%. Vascular plugs (Amplatzer™ Vascular Plug 6–10 mm, Abbott Cardiovascular, Plymouth, Minnesota, USA; *n* = 9) and detachable coils (*n* = 11) were the most frequently used devices. In most cases, coil embolization was performed utilizing a POD^®^ Coil (Penumbra Inc, Alameda, California, USA), which was sized 1:1 to the size of the artery, followed by Packing Coils (Penumbra Inc, Alameda, California, USA). Both techniques (vascular plug and coiling) were combined in nine cases (69.2%). Median platelet count pre-chemotherapy was 201 × 10(9)/L (range: 100–423 × 10(9)/L), platelet count nadir was 45 × 10(9)/L (range: 21–69 × 10(9)/L), and before PSAE platelet count was 82 × 10(9)/L (range: 45–171 × 10(9)/L). Median period from platelet nadir to the PSAE procedure was 78 days (range: 27–441 d). After PSAE, median platelet count increased significantly (110 × 10(9)/L; range: 68–169 × 10(9)/L) compared to pre-procedure platelet count (*p* = 0.027). Median period from PSAE to postinterventional assessment of platelet count was 7 days (range: 1–83 d). Median platelet count at the maximum at follow-up (median 94 days, range: 1–2470 d) was 191 × 10(9)/L (range: 31–518 × 10(9)/L). Platelet counts are displayed in Fig. 1. Chemotherapy was re-initiated after a median of 15 days (range 7–50 d). Splenic volume measurements were performed in nine patients, four patients did not have post-interventional contrast-enhanced CT angiogram or MRI. Mean pre-procedural splenic volume was 511.2 cm3 (*n* = 9, range 282–755 cm3). Post-procedural splenic volume at a median follow-up of 31 days (range 2–724 days) was 439.2 cm3 (*n* = 9, range 127–626 cm3). The difference between the pre- and post-procedural splenic volumes was not statistically significant (*p* = 0.121). The mean splenic infarction rate was 17.8% (range: 1.0–69.0%). An example case is shown in Fig. 2.

## Discussion

CIT has a crucial influence on cytotoxic therapy as well as the risk of adverse events in oncology patients requiring effective and rapid therapy. In this study, patients with CIT undergoing PSAE showed a significant increase of the platelet count without occurrence of adverse events or technical failure, followed by early re-initiation of chemotherapy. However, splenic volumes did not significantly decrease in size post-PSAE.

Besides platelet transfusions and the aforementioned medical CIT therapy, SAE is an effective and generally safe way to increase the platelet count. While both the proximal and distal SAE techniques are intended to permanently reduce splenic blood flow, residual perfusion in the terminal circulation areas are higher after PSAE due to the gastrointestinal collateral network (e.g., gastroepiploic artery). Following the limited evidence that PSAE has lower incidences of pain and other post-embolization adverse events compared to DSE, which also reflects the authors’ experience, we opted for the proximal embolization approach [22, 23].

PSAE for the management of thrombocytopenia has been reported in few studies with small patient cohorts. Bhatia et al. showed high technical success (100%) of PSAE in 13 patients with a significant increase of the post-procedural platelet count (209 × 10(9)/L; range: 83–363 × 10(9)/L) compared to the pre-procedural platelet count (88 × 10(9)/L; range:49–131 × 10(9)/L, *p* < 0.01) [15]. Average re-initiation of chemotherapy was 22 days, 30-day mortality was zero with no major adverse events, and splenic volume decreased significantly after PSAE (547 to 358 cm3, *p* < 0.01) with a mean splenic infarction rate of 29.5% [15]. Another study by Bhatia et al., investigating the long-term effects of PSAE, reported no significant alteration in mean platelet count within two weeks to three months after PSAE (*p* = 0.206) and no significant decrease in splenic volume after a mean follow-up of 733.57 days (*p* = 0.346), despite 100% technical success of PSAE in 14 patients [21]. Indication for PSAE was not limited to thrombocytopenia, but comprised a variety of reasons such as ascites, liver transplantation, splenomegaly and others. The heterogeneity of the study results could be due to the small number of patients and variable time points of assessment. In addition, different indications for PSAE with differing underlying pathological mechanisms could alter the therapeutic effect of the procedure, regarding both platelet count and splenic volumes.

Besides PSAE, DSE for thrombocytopenia is described to be effective in terms of technical success, increasing platelet counts and reducing splenic volumes. Ueda et al. reported a series of 45 patients undergoing DSE with gelatin sponge embolization for hypersplenism caused by liver cirrhosis, with a splenic infarction rate of 50.9% and a pre-/post-DSE platelet count of 57 × 10(9)/L and 96 × 10(9)/L, respectively (*p* = 0.001) [19]. Rabczynksi et al. analyzed DSE for thrombocytopenia in 22 patients with an peri-procedural increase of the mean platelet count from 22.0 × 10(9)/L to 87.7 × 10(9)/L (*p* < 0.05) in a mean period of 194 days [20]. Notably, one adverse event requiring splenectomy (‘*fluid reservoir in the spleen*’) and two events of severe post-embolization syndrome (including pain, fever, nausea, vomiting and inflammatory parameter increase) occurred, all associated with increased length of hospital stay [20].

Only a few studies compared PSAE to DSE for non-chemotherapy related thrombocytopenia. Hill et al. reported both PSAE (*n* = 14) and DSE (*n* = 84) procedures in one study for 98 patients with hypersplenism-related thrombocytopenia, resulting in 59% of the patients with post-SAE platelet counts over 100 × 10(9)/L and 41% of the patients not experiencing recurrence of thrombocytopenia [26]. Overall, 30-day-mortality was 7% (2% of procedure-related mortality due to bacteremia/sepsis and pneumonia), and overall major adverse event rate was 8%, with readmission for persistent post-embolization syndrome. Proximal versus distal SAE showed no difference regarding platelet elevation twelve months after intervention (*p* = 1.0) [26]. A study with the purpose of comparing proximal versus distal SAE has been performed for patients with vascular injuries including a total of 202 patients (PSAE: *n* = 64, 31.7%; DSE: *n* = 84, 41.6%; combined: *n* = 54, 26.7%) [27]. No significant difference regarding clinical success was found, considering an overall hemostasis success rates of 92.6% and subgroup success of 93.8% (PSAE), 88.1% (DSE) and 98.1% (combined, *p* = 0.079). Splenic abscess occurred in six patients, of which five received DSE and one combined SAE (*p* = 0.092) [27]. To date, no studies have compared PSAE to DSE for chemotherapy induced thrombocytopenia.

In addition to thrombocytopenia, SAE is a common treatment strategy for traumatic injuries, given the fact that the spleen is the most commonly injured organ in blunt abdominal trauma [13]. It is indicated for stable patients with moderate to severe splenic injuries. Both PSAE and DSE are eligible approaches, whereas PSAE is favored for multifocal injuries. A typically faster procedure time can be an advantage of PSAE in the management of trauma patients, whereas potential rebleeding from collaterals can be a disadvantage [13]. In a retrospective multicenter trial including 1275 patients, SAE significantly increased the likelihood of splenic salvage compared to nonoperative management without SAE of blunt splenic injuries (OR: 5, 95% CI 1.8–13.5) [30].

There are limitations to this study, most notably the small patient number. Furthermore, this is a single-center retrospective study with possible reporting deficits and without control group, which might lead to selection bias and confounding variables. Timing of platelet count assessment as well as splenic volume assessment varies, which could have a relevant effect on respective variables. Pre-PSAE platelet count of one patient in this study was relatively high with 171 × 10(9)/L, following an initial chemotherapy related platelet drop from 170 × 10(9)/L to 52 × 10(9)/L. This patient remained asymptomatic regarding CIT and did not receive a platelet transfusion. No other explanation than an interval of two months between the nadir and the pre-PSAE platelet count, during which the patient did not receive chemotherapy, was found. The maximum platelet count, recorded throughout the entire available follow-up period for each patient presents a wide range (median 94 d, range 1-2470d), due to the already mentioned limitations regarding not-standardized follow-up regimen. Assessment of this outcome was intended to illustrate the general long-term trend; any further long-term changes to the chemotherapy regimen (besides re-initiation) were not entirely considered, nor were other factors influencing platelet counts. For this reason, no statistical analysis was performed in this regard, and therefore no conclusions can be drawn regarding the long-term effect of PSAE. Assessment of pre-procedural splenic volume of one patient in this study was performed with the latest available CT scan six month prior to SAE, which could explain the increase of his splenic volume from 298.3 cm3 to 410.5 cm3 despite the intervention and corresponding impacts on mean splenic volumes. Future studies with larger sample sizes are needed to investigate long-term therapy effects and optimize patient selection for PSAE. Prospective randomized controlled studies comparing proximal and distal SAE techniques could help to improve control of adverse events such as symptoms of the post-embolization syndrome and provide better knowledge of individualized CIT treatment. In these studies, timing of assessment (platelet count and splenic volumes) should be standardized.

## Conclusion

Proximal SAE is a safe and effective treatment option in patients with CIT, with potential benefits in early re-initiation of chemotherapy. Further studies should be conducted to evaluate the longer-term effects of the procedure, further identify eligible patients, and compare to distal SAE.

## Data Availability

The data that support the findings of this study are available on request from the corresponding author.
